# Evaluation of Allergic Conjunctivitis Prevalence and Attitude Toward Prevention and Control in Saudi Arabia

**DOI:** 10.7759/cureus.57711

**Published:** 2024-04-06

**Authors:** Mahadi Bashir, Twfiq A Alghamdi, Ayman M Alzahrani, Mohammed Ahmed A Alghamdi, Shoog K Aloleeit, Zainab AlHajji, Essa S Alsultan, Nour H Aljamaan, Liyan K Abu Rukbah

**Affiliations:** 1 Ophthalmology, Al-Baha University, Al-Baha, SAU; 2 College of Medicine, Al-Baha University, Al-Baha, SAU; 3 College of Medicine, King Faisal University, Al-Hofuf, SAU; 4 College of Medicine, Taif University, Taif, SAU

**Keywords:** prevention, knowledge attitude, prevalence, allergic conjunctivitis, ophthalmology

## Abstract

Background: Allergic conjunctivitis (AC) is a prevalent ocular condition with a substantial impact on individuals' quality of life. This study aimed to explore the demographic patterns, prevalence, symptoms, awareness, and attitudes associated with AC, while also examining potential associations with gender, age, and region of residence in Saudi Arabia.

Methods: A cross-sectional study was conducted involving 487 participants. Data was collected through a self-administered questionnaire that included demographic information about AC prevalence, symptoms, attitudes, and awareness levels. Statistical analyses, including chi-square tests, were employed to examine associations between variables.

Results: The study revealed a prevalence of AC (89, 18.3%) with common symptoms being eye redness (73, 82%) and itching (73, 82%). Participants displayed diverse awareness levels, with (376, 77.2%) correctly defining AC. The majority demonstrated either a good (230, 47.2%) or insufficient (196, 40.2%) attitude, while 54 (11.1%) had a sufficient attitude, and 7 (1.4%) exhibited an excellent attitude and awareness. Significant regional disparities were observed, impacting both prevalence and attitudes. While no gender differences were noted, the age group of 31-40 displayed a higher prevalence.

Conclusion: In this study, among 487 participants, the prevalence of AC was found to be 89 (18.3%). Meanwhile, attitude levels varied, with the majority demonstrating either a good or insufficient attitude. This provides valuable insights into the prevalence, symptoms, and awareness of AC in our population. The regional disparities underscore the need for tailored interventions addressing specific geographical contexts. The findings contribute to the broader understanding of AC, emphasizing the importance of targeted education and regional considerations in managing and preventing this condition.

## Introduction

Ocular allergy (OA), also known as allergic conjunctivitis (AC), is a wide category including a variety of illnesses in which an abnormal conjunctival inflammation occurs in reaction to allergens that are typically innocuous [1]. It is one of the most common ocular surface conditions treated by primary eye care professionals [2].

While providing healthcare to those with chronic conditions, the WHO has emphasized the importance of patients and their families. In the world today, the incidence of allergies is a prevalent chronic ailment that is significantly rising [3]. The symptoms of AC vary widely, can be severe, have an adverse effect on quality of life, and even pose a threat to vision [4]. Air pollution was identified as one of the hypothesized related factors that not only aggravated the symptoms but also increased the prevalence of its severe forms [5]. A current estimate is that 20% of the world's population suffers from allergies, and allergy rates are rising. Up to 40-60% of allergy sufferers experience eye symptoms [6]. Children and adults can both be affected by AC, which frequently coexists with other allergic illnesses, including asthma, atopic dermatitis, or food allergies; however, it is particularly linked to allergic rhinitis [7].

Close cooperation between the ophthalmologist and the allergist is necessary for the investigation and treatment of AC, especially in a department with experts in both domains [8]. Additionally, the patient should avoid allergens that could trigger their other allergies [9]. Antihistamines, mast cell stabilizers, and non-steroidal anti-inflammatory drugs are effective topical treatments for reducing the bothersome symptoms of burning, itching, tearing, and conjunctival erythema without causing significant effects, and when using topical steroids to treat severe OA symptoms, it is occasionally necessary to be aware of their recognized long-term side effects [10]. The aim of this study is the evaluation of AC prevalence and attitude toward prevention and control in the Kingdom of Saudi Arabia.

## Materials and methods

The present study adopted a cross-sectional descriptive design to investigate the attitudes toward AC and the corresponding preventive measures among individuals residing in Saudi Arabia. The sample size was determined using the Raosoft calculator, resulting in a minimum sample size of 385 with a 95% confidence level and a 5% margin of error.

The inclusion criteria comprised individuals of both genders, aged 18 and above, residing in Saudi Arabia. Conversely, exclusion criteria encompassed individuals below 18 years of age, those not residing in Saudi Arabia, and those unwilling to participate in the study. These criteria were established to ensure the relevance and applicability of the findings to the targeted population.

Data collection was conducted through a self-administered questionnaire over a period from January 2024 to February 2024. The questionnaire was divided into four components. Informed permission is followed by a survey of participant demographics, a prevalence assessment, and multiple questions intended to gauge the general understanding of AC and attitudes toward the knowledge of preventive and control measures.

Upon completion of the data collection phase, the gathered information was entered into Microsoft Excel (2016). Subsequently, the data was imported into the IBM SPSS Statistics for Windows, Version 20 (Released 2011; IBM Corp., Armonk, New York, United States) for comprehensive statistical analysis. This rigorous analytical approach allowed for a thorough exploration of patterns, trends, and correlations within the collected data. In the tables, alongside the p-value, the corresponding t-value, chi-square value, and F-value derived from the t-test, chi-square test, and analysis of variance, respectively, as applicable, were included.

A scoring system was devised to quantify a participant's attitude, with each question contributing one point to a total of 20 points. The scoring categories were established as follows: 0/20 indicated poor attitude and awareness, 1-10 denoted insufficient attitude, 10-15 represented a good attitude, 15-20 indicated a sufficient attitude and a perfect score of 20/20 reflected excellent attitude and awareness. This scoring system provided a nuanced and quantifiable assessment of participant's attitudes toward AC.

Furthermore, ethical approval for the study was obtained from the Institutional Review Board (IRB) of Al Baha University Faculty of Medicine under the reference REC/SUR/BU-FM/2023/76, ensuring compliance with ethical standards and safeguarding the rights and well-being of the study participants. This approval underscored the commitment to conducting research in an ethical and responsible manner.

## Results

An overview of the demographic characteristics of the study participants is provided in Table 1. A total of 487 individuals participated in the study, with 166 (34.1%) being male and 321 (65.9%) female. The majority of participants fell within the age range of 18-30 years (324, 66.5%), and the distribution across regions was as follows: Northern region (17, 3.5%), Western region (232, 47.6%), Eastern region (88, 18.1%), Southern region (109, 22.4%), and Central region (41, 8.4%).

**Table 1:** The demographic factors of the participants

		Count	Percentage
Gender	Male	166	34.1%
	Female	321	65.9%
Age	18-30	324	66.5%
	31-40	50	10.3%
	41-50	75	15.4%
	51-60	33	6.8%
	61-70	5	1.0%
Region	Northern region	17	3.5%
	Western region	232	47.6%
	Eastern region	88	18.1%
	Southern region	109	22.4%
	Central region	41	8.4%

The prevalence of self-reported diagnoses of AC among the total respondents is presented in Table 2. A total of 89 (18.3%) reported having experienced AC. The most common symptoms reported were eye redness (73, 82.0%) and eye itching (73, 82.0%). Regarding treatment, 35 (39.3%) participants reported using no treatment, while antihistamines (31, 34.8%) and antibiotics (22, 24.7%) were the most commonly used interventions. The analysis reveals that there is no statistically significant difference in the prevalence of AC between genders (p = 0.681). Both males and females exhibit relatively similar rates, with 134 (80.7%) males and 264 (82.2%) females reporting no history of AC. Similarly, when examining age groups, the difference in prevalence is not statistically significant (p = 0.115). Although there are variations across age categories, ranging from 2 (40.0%) to 44 (88.0%), the overall prevalence does not exhibit a clear age-related trend. However, a noteworthy finding emerges when considering the region of residence. A significant association is observed (p = 0.002), indicating regional disparities in the prevalence of AC. Participants from the Central region report a lower prevalence (8, 19.5%) compared to those in the Eastern region (26, 11.2%) and other regions.

**Table 2:** The prevalence of self-reported diagnoses of allergic conjunctivitis, symptoms, and treatment

		Count	Percentage
Have you ever had allergic conjunctivitis?	No	398	81.7%
	Yes	89	18.3%
What were the associated symptoms?	Eye redness	73	82.0%
	Eye pain	47	52.8%
	Eye itching	73	82.0%
	Others: eye swelling and tears	1	1.1%
What kind of drug was taken?	None	35	39.3%
	Antihistamines	31	34.8%
	Antibiotics	22	24.7%
	Analgesics	19	21.3%

The awareness and attitudes of participants regarding AC are discussed, and the results are presented in Table 3. The majority of participants (376, 77.2%) accurately identified AC as eye inflammation resulting from an allergic reaction to a trigger. However, a notable portion (76, 15.6%) misinterpreted it as an eye infection due to bacteria. Participants displayed varying awareness of common triggers for AC. A significant portion correctly identified pollen (172, 35.3%), dust mites or insects (50, 10.3%), and pet dander (61, 12.5%) as common triggers. In terms of treatment, a notable proportion correctly identified antihistamines (164, 33.7%) as a common remedy, while misconceptions were observed with (109, 22.4%) associating moisturizing drops as a treatment. The majority of participants (384, 78.9%) correctly acknowledged the avoidance of allergens as the primary preventive measure for AC.

**Table 3:** The awareness and attitude of the participants toward conjunctivitis control

Items	Correct choice	Count	Percentage
Definition of allergic conjunctivitis	Eye inflammation due to an allergic reaction to a trigger	376	77.2%
Common symptoms of allergic conjunctivitis	Eye pain, itching, and redness	101	20.7%
Not a common trigger of allergic conjunctivitis	Bacterial infection	204	41.9%
Contagious in nature	False	197	40.5%
Commonly used drug in this case	Antihistamines	164	33.7%
Definitive preventive measure	Avoid allergens	384	78.9%
Contact lens wearers are at a higher risk	True	270	55.4%
Reducing exposure to allergens indoor	Using air purifiers inside the home and freshening the air regularly	393	80.7%
Avoid rubbing the eyes is an important preventive measure	True	363	74.5%
Not a recommended method for reducing exposure to dust mites	High humidity level in the bedroom	207	42.5%
Preventing pet allergies	Regular bathing and drying of pets	175	35.9%
Antihistamines can reduce the symptoms	True	234	48%
Important preventive methods in children	Regular hand washing	397	81.5%
Not a recommended action for allergic conjunctivitis	Frequent eye rubbing	326	66.9%
Cold compresses could help reduce the symptoms	True	313	64.3%
Preventing indoor allergens	Cleaning and moping surfaces inside the house regularly	377	77.4%
Preventing outdoor eye allergies	Wearing sunglasses	271	55.6%
Complications of untreated chronic conjunctivitis	Corneal abrasion	123	25.3%
Not a recommended behavior	Taking medications or herbs before seeking medical advice	279	57.3%
Management of allergic conjunctivitis by avoiding exposure to allergies and using appropriate medications	Agree and strongly agree	369	75.8%

**Figure 1:**
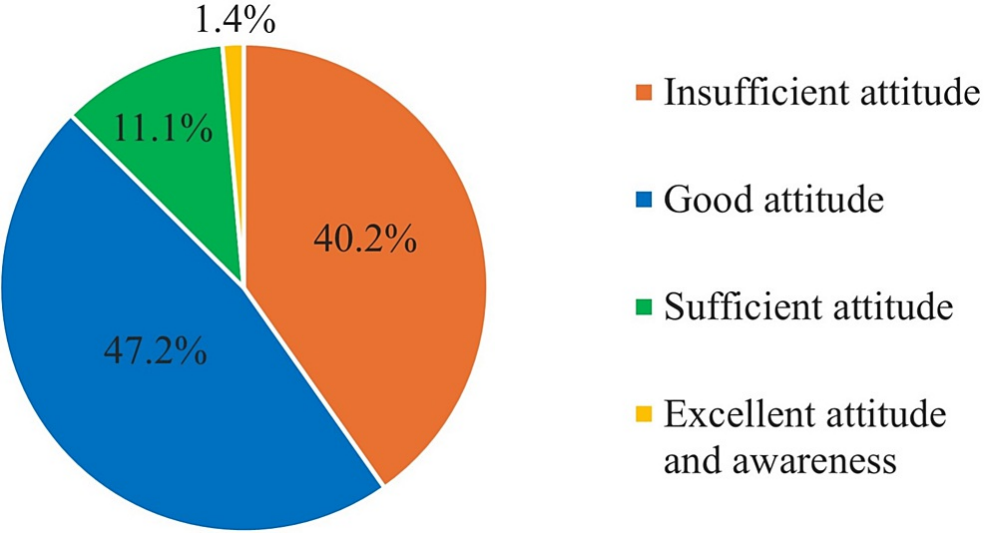
The attitude level among the participants

When categorizing the participants based on their attitude levels, this is shown in Figure 1. The majority demonstrated either a good (230, 47.2%) or insufficient (196, 40.2%) attitude, while others (54, 11.1%) had a sufficient attitude, and (7, 1.4%) exhibited an excellent attitude and awareness toward AC.

The association between attitude level and demographic factors can be explored, as shown in Table 4. The analysis indicates that there is no significant difference in attitude levels between genders (p = 0.096). Both males and females display similar distributions across attitude categories. However, when considering age groups, again, no significant differences are observed (p = 0.796). Attitude levels are relatively consistent across different age categories. Notably, the region of residence demonstrates a significant association with attitude levels (p = 0.000), highlighting regional disparities in attitudes toward AC. Participants from the Northern region exhibit a distinct attitude pattern compared to other regions. When examining the relationship between a history of AC and attitude levels, no significant association is found (p = 0.062), suggesting that the presence of AC does not strongly correlate with specific attitudes.

**Table 4:** The association between attitude level and demographic factors

		Attitude level								
		Insufficient attitude		Good attitude		Sufficient attitude		Excellent attitude and awareness.		Chi-square value, p-value
		Count	Percentage	Count	Percentage	Count	Percentage	Count	Percentage	
Gender	Male	76	45.8%	72	43.4%	18	10.8%	0	0.0%	6.334, 0.096
	Female	120	37.4%	158	49.2%	36	11.2%	7	2.2%	
Age	18-30	133	41.0%	149	46.0%	39	12.0%	3	0.9%	7.866, 0.796
	31-40	21	42.0%	22	44.0%	5	10.0%	2	4.0%	
	41-50	30	40.0%	37	49.3%	6	8.0%	2	2.7%	
	51-60	10	30.3%	19	57.6%	4	12.1%	0	0.0%	
	61-70	2	40.0%	3	60.0%	0	0.0%	0	0.0%	
Region	Northern region	7	41.2%	7	41.2%	2	11.8%	1	5.9%	36.498, 0.000
	Western region	98	42.2%	113	48.7%	20	8.6%	1	0.4%	
	Eastern region	35	39.8%	29	33.0%	21	23.9%	3	3.4%	
	Southern region	37	33.9%	64	58.7%	8	7.3%	0	0.0%	
	Central region	19	46.3%	17	41.5%	3	7.3%	2	4.9%	
Have you ever had allergic conjunctivitis?	No	170	42.7%	183	46.0%	39	9.8%	6	1.5%	7.351, 0.062
	Yes	26	29.2%	47	52.8%	15	16.9%	1	1.1%	

## Discussion

The findings of this investigation offer insightful information about the participants' demographics, prevalence, awareness, and attitudes toward AC. This discussion will explore key aspects of the results, including demographic patterns, prevalence rates, awareness levels, and the association between demographic factors and attitudes toward AC.

The results of the current study among 487 participants shed light on the prevalence of self-reported AC, symptoms, and treatment patterns. The reported prevalence of AC among participants is 89 (18.3%). The prevalence in our study is consistent with global estimates. Two studies by Berger et al., and Leonardi et al., reported that the prevalence of AC ranges between 6% and 30% [11,12]. In addition, another two studies, conducted in Iran and Denmark, reported a prevalence of 15.9% and 15% among the adult population [13,14]. However, some other studies reported a lower prevalence of AC, including the study of Kang et al., among the adult Korean population, which reported a prevalence of 5.6% [15], and another study in India among children, where the prevalence was 8.5% [16]. In Saudi Arabia, a previous study conducted by Alqurashi et al. found that among 2187 adult participants in the Western region, there was a significantly higher prevalence of 70.5% [17], while in the Taif region, another study reported a prevalence of 3.4% which is significantly lower than our result [18]. This figure underscores the significance of the condition in the study population, indicating a considerable number of individuals affected.

The most commonly reported symptoms include eye redness (73, 82%) and eye itching (73, 82%), aligning with typical AC symptoms. The reported symptoms, especially eye redness and itching, mirror established literature documenting these as hallmark features of AC [19-21]. Understanding the prevalence and symptoms provides a foundation for assessing the burden of the condition and informing public health strategies.

The accurate identification of AC as eye inflammation due to allergic reactions [22] by 376 (77.2%) participants is in line with studies emphasizing the importance of raising awareness about the condition. The observed misconceptions about triggers and treatment echo findings from previous studies [23,24], emphasizing the persistence of misinformation and the necessity for targeted educational interventions. The positive trend in recognizing antihistamine drops (164, 33.7%) aligns with the literature advocating for the use of antihistamines as a primary treatment modality [4,25]. The emphasis on avoiding allergens (384, 78.9%) as a preventive measure corresponds with established guidelines promoting allergen avoidance strategies [26].

The lack of significant gender differences in prevalence aligns with studies suggesting that gender may not be a major risk factor for AC [17,27]. While there is no significant difference between genders, regional variations are evident.

The regional disparities observed in this study are consistent with research highlighting variations in allergic conditions based on geographical and environmental factors [28]. This regional disparity emphasizes the need for region-specific interventions and suggests potential environmental factors contributing to the prevalence differences.

It is crucial to acknowledge the limitations of the study, such as potential recall bias in self-reported data and the need for more diverse participant representation. Recommendations for future research include longitudinal studies to track changes in awareness and attitudes over time and explore the impact of specific interventions on improving knowledge.

## Conclusions

In conclusion, the study of 487 individuals showed a (89, 18.3%) prevalence of AC. In the meantime, there was a range in attitude levels, with most people exhibiting either an adequate or an inadequate attitude. This offers important information about AC awareness, symptoms, and prevalence in our community. The regional differences highlight the necessity of specialized solutions that take into account unique geographic circumstances. The results emphasize the value of focused education and local factors in the management and prevention of AC, adding to our understanding of the problem.
